# Data of macroinvertebrates assemblage across different stretches of an urban Palmiet River in Durban, South Africa

**DOI:** 10.1016/j.dib.2021.107493

**Published:** 2021-10-20

**Authors:** J. Lebepe, N. Khumalo, A. Mnguni, S. Pillay, S. Mdluli

**Affiliations:** School of Life Sciences, University of KwaZulu-Natal, Private Bag x54001, Durban 4000, South Africa

**Keywords:** Urban River, Water pollution, Anthropogenic litter, Integrated habitat assessment score, Amphipoda, Notonemouridae

## Abstract

Urban rivers have been overlooked as they are regarded as unnatural with poor ecological conditions to support aquatic life. This dataset presents the abundance and taxa richness of macroinvertebrates collected in an urban Palmiet River, which showed highly variable stretches with respect to water quality and physical habitat availability. A YSI 556 MPS handheld multiparameter instrument was used to measure physical variables of the water, whereas samples were taken using sampling bottles and kept in the fridge prior to nutrient analysis. Habitat assessment was carried out following the integrated habitat assessment score (IHAS) protocol. Macroinvertebrates were collected using modified SASS5 protocol, where stone, vegetation, and gravel sand, and mud biotopes were sampled. Macroinvertebrates were identified to family levels, and abundance and taxa richness were calculated. This data affirms the capacity of urban rivers to harbor aquatic biota and to self-purify along the longitudinal gradient. The data further attest that the response of urban rivers to anthropogenic activities does not differ from natural streams, and the assemblage of macroinvertebrates is driven by water quality and physical habitat. Moreover, the role played by anthropogenic litter in the absence of natural habitat is stressed. Lastly, this data can guide urban ecologists when designing studies for highly variable urban river systems as it illustrates the dynamics of urban ecosystems and their potential to harbor aquatic biota.

## Specifications Table

**Table:** 

Subject	Environmental Science (Aquatic Ecology)
Specific subject area	Aquatic macroinvertebrates in an urban lotic water system
Type of data	Figures Table
How the data were acquired	The data was acquired through field sampling, which was conducted seasonally from March 2017 to January 2018. Habitat quality was assessed following McMillan (1998) protocol. Macroinvertebrates and water were collected following protocol adapted from Dickens and Graham (2002), and identification was carried out with the aid of Gerber and Gabriel (2002a,b) illustration and field guides. A magnifying glass was used for macroinvertebrates identification in the field, with a dissecting microscope being used in the lab. Physical water variables were measured using YSI 556 MPS handheld multiparameter instrument. An Ion Chromatography System (ICS-5000 Single Pump) with a Suppressor and Conductivity Detector was used for measuring nutrients in the water.
Data format	Raw Analyzed Filtered
Description of data collection	This data was collected seasonally to cover different climatic conditions. Sampling sites were selected based on their location relative to various land use (Fig. 1).
Data source location	• City/Town/Region: Durban in Pinetown • Country: South Africa • Latitude and longitude (and GPS coordinates) for the sites: • Site 1: 29°49′17″S, 30°57′12″E • Site 2: 29°49′25″S, 30°55′33″E • Site 3: 29°49′50″S, 30°54′37″E • Site 4: 29°48′53″S, 30°52′55″E • Site 5: 29°48′34″S, 30°52′11″E • Site 6: 29°47′56″S, 30°51′14″E
Data accessibility	Repository Name: Mendeley Data DOI: https://doi.org/10.17632/stgy64rb9z.1

## Value of the Data

• This data gives an idea of how macroinvertebrates may differ along the longitudinal gradient of an urban river characterized by highly variable stretches and how important could anthropogenic litter be in modified stretches.
• This data may benefit authorities responsible for biodiversity conservation in urban rivers as it attests to the capability of these systems in harboring aquatic biota.
• This data can guide urban ecologists when designing studies for highly variable urban river systems as it illustrates the dynamics of urban ecosystems and their potential to harbor aquatic biota.
• This data can also be of particular importance as it stresses the role played by anthropogenic litter in providing additional habitat in stretches characterized by poor physical habitat quality.

## 1 Data Description

Complete metadata presenting the levels of pH, water temperature, DO, salinity, TDS, NO_2_, NO_3_, NH_3_, SO_4_, and P in the water column, habitat scores, and macroinvertebrates assemblage in relation to sites and seasons is hosted in Mendeley data [1]. Relationships between water variables are presented in Fig. 2; nevertheless, NO_2_ level was below detection limit during most samplings, hence, it was not included in correlation analysis. Furthermore, asterisks in Fig. 2 are denoting significant relationships. Fig. 3 presents log-transformed taxa abundance across 6 sites, with dark blue denoting fewer to absence whereas light blue denote higher abundance. A non-metric multidimensional scaling plot shows the variation of macroinvertebrates assemblage across 6 sites using Euclidean distance (Fig. 4). Fig. 5 presents calculated scores for the South African Scoring System (SASS5) vs Average Score Per Taxon (ASPT) observed at 6 sampling sites along the Palmiet River. The association between SASS score and ASPT is also depicted in Fig. 5.

## 2 Experimental Design, Materials and Methods

### 2.1 Experimental design

Sampling was conducted at 6 sites in the Palmiet River. Site 1 was a few kilometers downstream of the Palmiet Nature Reserve, Site 2 was within the Palmiet Nature Reserve, whereas Site 3 was a few kilometers upstream of the Palmiet Nature Reserve. Site 4 was further upstream, which was situated downstream the industrial area, Site 5 within the industrial area, and Site 6 in the headwaters of the Palmiet River, which was bordered by a residential area.

### 2.2 Study area

**Fig. 1:**
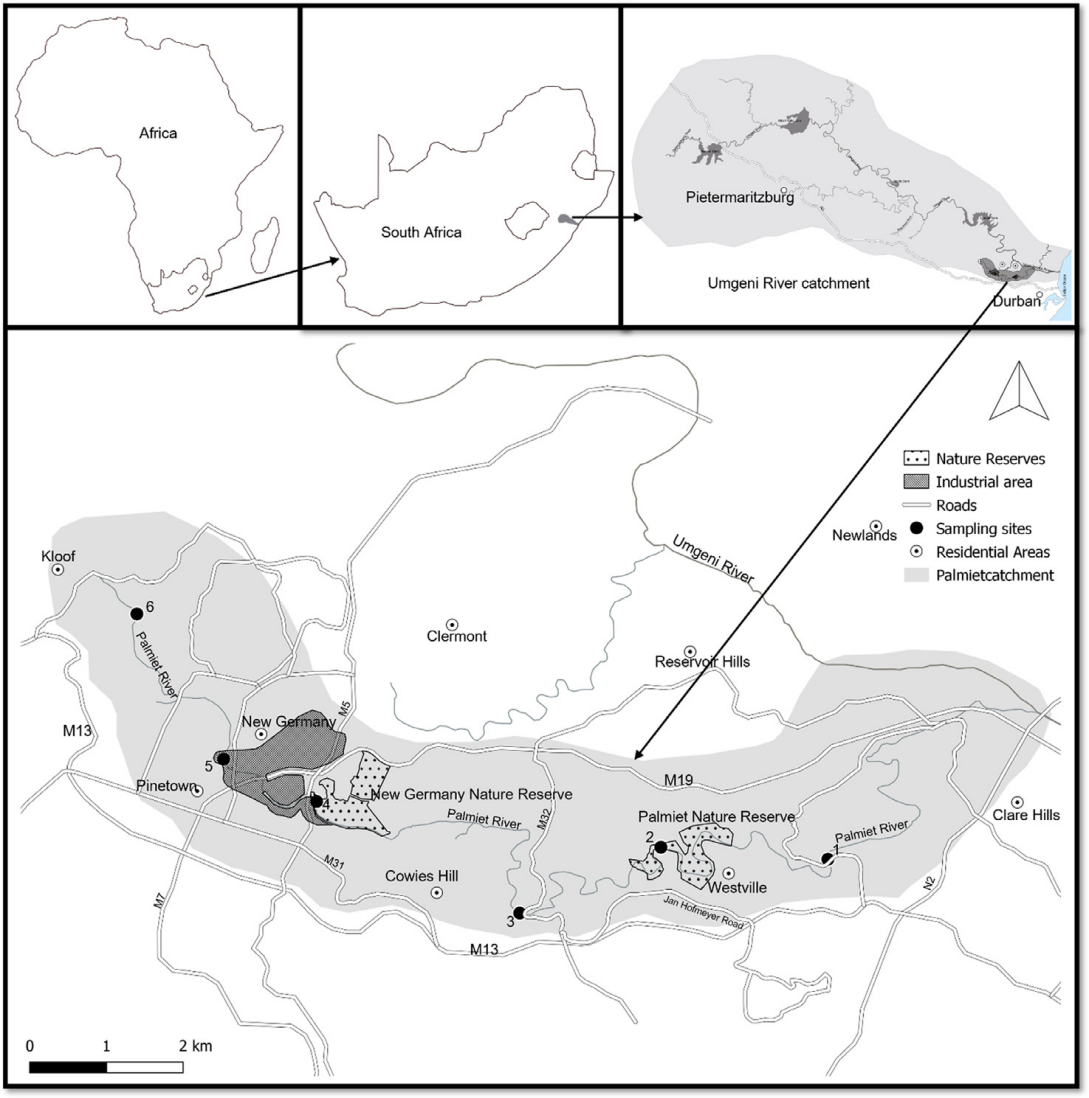
The Palmiet River catchment with black dots denoting 6 sampling sites.

The Palmiet River is bordered by a residential area in the headwaters, an industrial area in the middle, and another residential area in the lower stretch. The lower stretch of the river also runs through the Palmiet Nature Reserve [2]. The Palmiet River originates from the Kloof escarpment, and it joins the Umgeni River system before the latter empties into the Indian Ocean (Fig. 1). The river is characterized by highly variable stretches with regard to the river bed, stream width, riparian vegetation, aquatic biota physical habitat, and water quality along the longitudinal gradient [3,4].

**Fig. 2:**
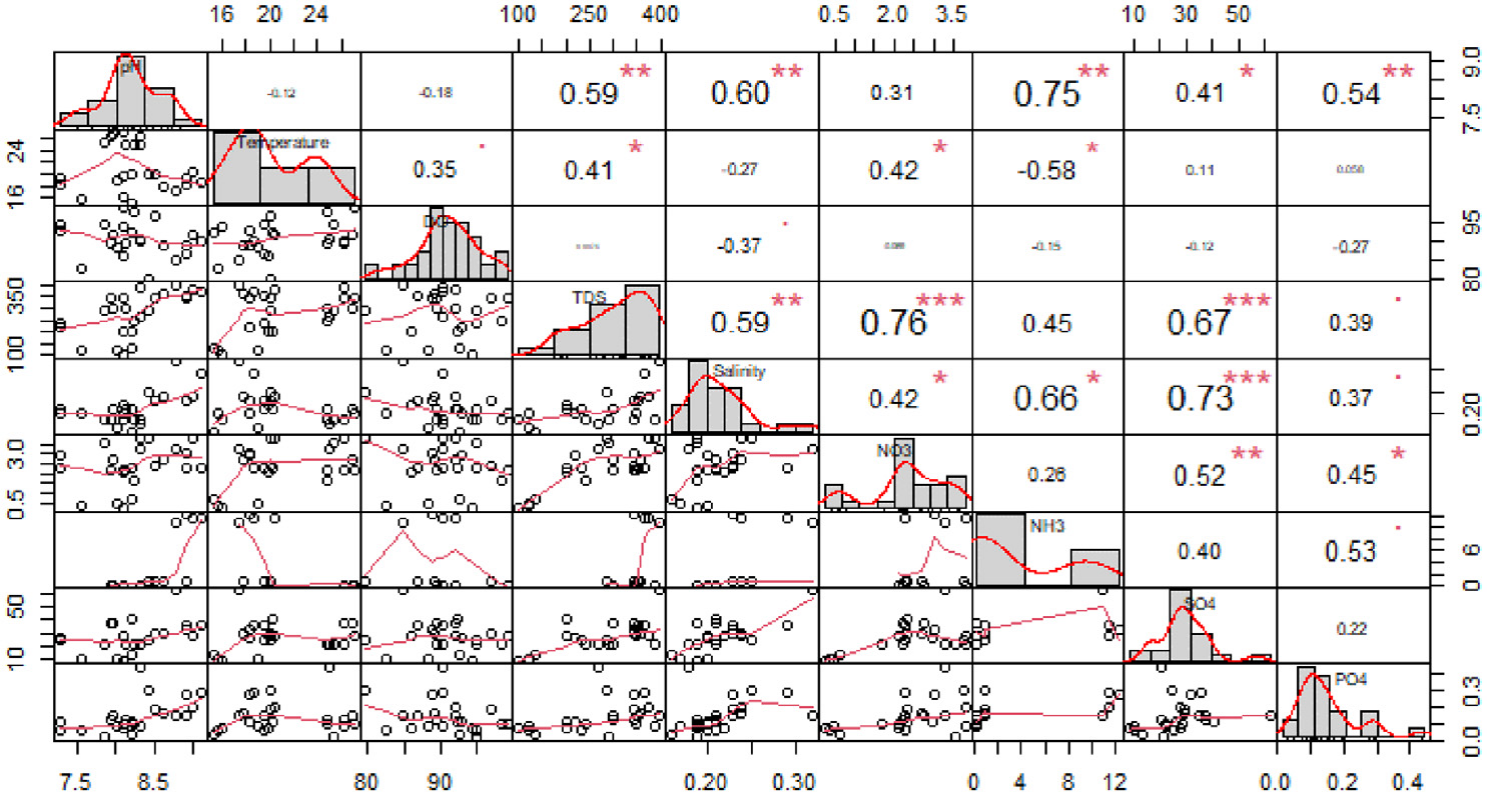
Correlation coefficients for water quality variables observed along an urban Palmiet River.

**Fig. 3:**
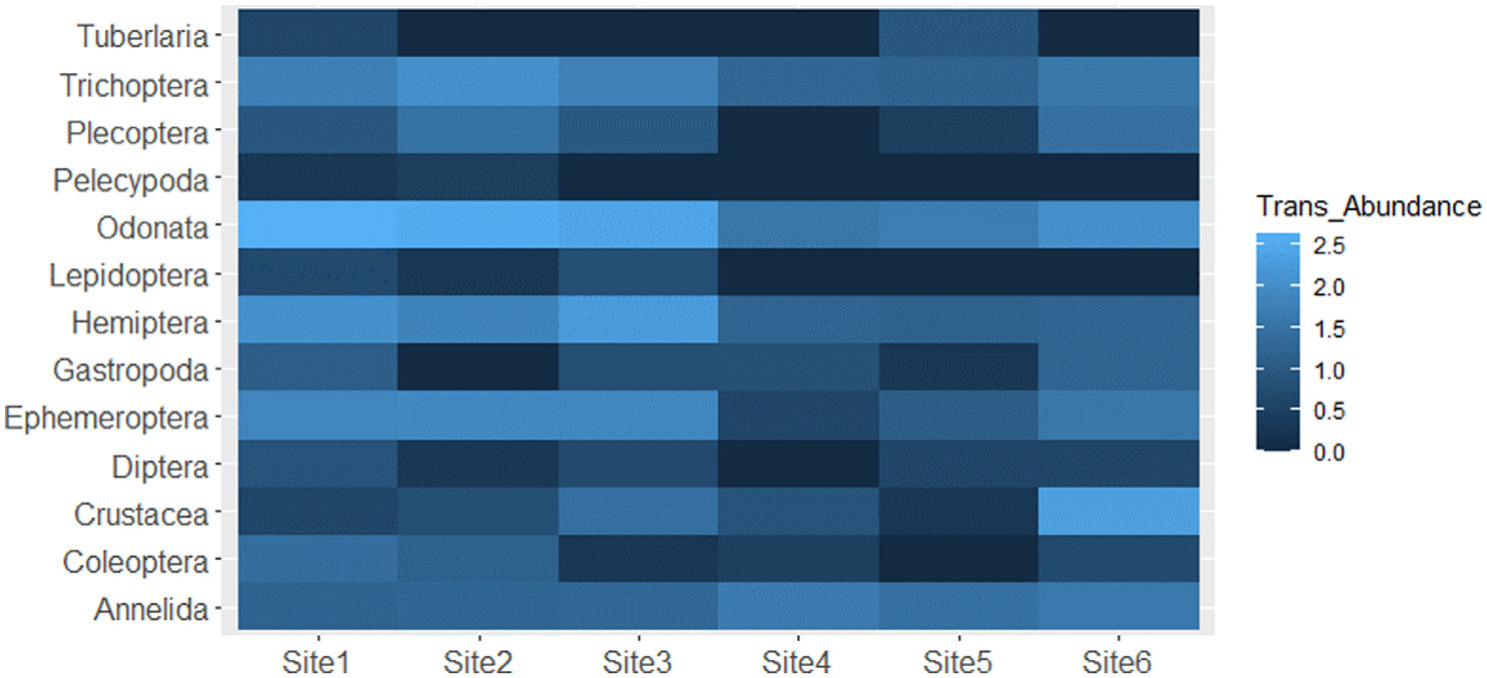
Log-transformed taxa abundance observed across 6 sites in an urban Palmiet River.

**Fig. 4:**
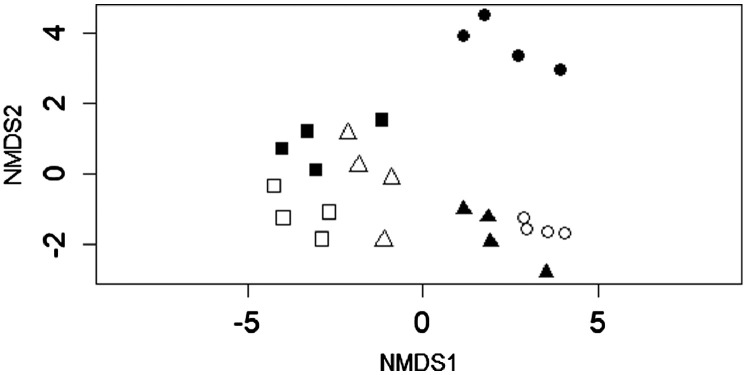
A non-metric multidimensional scaling plot ordinating macroinvertebrates assemblage Site 1 (□), Site 2 (■), Site 3 (△), Site 4 (▴), Site 5 (○), Site 6 (●) in an urban Palmiet River.

**Fig. 5:**
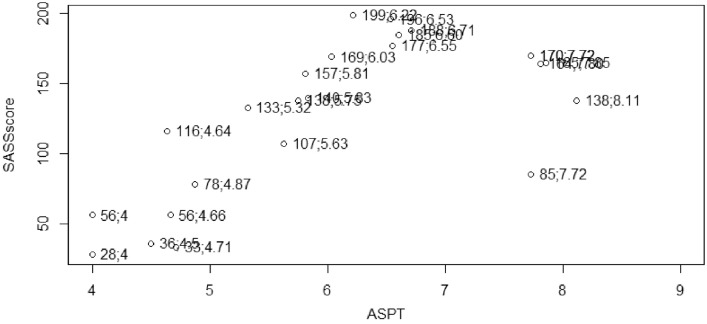
South African Scoring System vs Average score per taxon (SASSscore;ASPT) recorded across 6 sites in an urban Palmiet River.

### 2.3 Water sampling and analysis

Sampling was conducted seasonally from March 2017 (Autumn) to January 2018 (Summer) across 6 sites. The water temperature, dissolved oxygen (DO), pH, total dissolved solids (TDS) and salinity were measured using a YSI 556 MPS handheld multiparameter instrument. The water samples were collected from each side using an acid-pretreated bottle kept in a cooler box and later transferred to a fridge upon arrival to the School of Life Sciences laboratory of the University of KwaZulu Natal. The water was syringe-filtered through a 0.45 µm pored membrane. The nutrients analysis in the water was done at an accredited laboratory following APHA [5] protocol using an Ion Chromatography System (ICS-5000 Single Pump) with a Suppressor and Conductivity Detector. The detection limits were 0.1 mg/l for nitrogen components and sulfate and 0.01 mg/l for Phosphate.

### 2.4 Habitat assessment

Habitat was assessed using protocol adapted from McMillan [6]. Scores were assigned to each component of the habitat based on the condition. The sampling habitat was divided into three components, stone in current (20%), vegetation (15%), and other habitats (20), which all constituted 55% to the overall habitat score (Appendix S1). Other habitats component was comprised of stone out of current (SOC), bedrocks, gravel sand, and mud (GSM), and anthropogenic litter such as pipes, plastics, tins etc. Stream characteristics which included attributes such as river width, water depth, water color etc. constituted 45% to the overall habitat score (Appendix S1). The Total Habitat score was calculated as the sum of final scores for each sampling habitat category (55%), whereas the Total IHAS score was the sum of Total Habitat (55%) and Stream Condition (45%) scores. The Palmiet River is an urban stream with some stretches exhibiting a high accumulation of anthropogenic solid waste materials; therefore, the anthropogenic litter and anthropogenic litter effect components were included under the Stream Condition. Anthropogenic litter was assigned a score of 0 where absent, 2 if similar items were observed, and 4 for a mixture of items. Anthropogenic effect on water flow was assigned a score of 2 if there was a severe effect (notable influence on water velocity) and 4 if there was no effect.

### 2.5 Macroinvertebrates

Macroinvertebrates were collected seasonally at 6 sites along the longitudinal gradient of the Palmiet River (Fig. 1). Sampling was carried out following a protocol adapted from Dickens and Graham (2002). Different biotopes, aquatic vegetation, stones, and gravel, sand, and mud (GSM) were sampled at each site. Samples from marginal and aquatic vegetations were pulled together and treated as a vegetation biotope. For the stone biotope, specimens collected from the bedrock, stone in and out of current were treated as one entity as a stone biotope. Specimens from gravel, sand, and mud were pooled together and treated as a GSM biotope.

For the stone biotope, the bottom substrate was disturbed for a minimum of 3 min but not exceeding 5 min, and a net was placed about 0.5m downstream of the disturbed area. Some stones were picked up and scrapped for those inhabiting underneath. For GSM biotope, the net was placed about a meter downstream of the disturbed area to allow sand to sink before it gets into the net. For vegetation biotope, the net was pushed repeatedly against the vegetation for approximately 2 meters to dislodge the attached organisms, and this continued for a minimum of 3 min but not exceeding 5 min. Everything captured by the net was transferred to a white tray. Organisms were identified to family levels using illustration and field guides by Gerber and Gabriel [7] and Gerber and Gabriel [8]. A magnifying glass was used in the field. Some macroinvertebrates were preserved in 70% ethanol for quality control in the lab and auditing in the laboratory. South African Scoring System (SASS5) scores were calculated during each sampling, and the Average Score Per Taxon (ASPT) was calculated to determine the ecological health of the river stretches.

## Ethics Statements

Not applicable.

## CRediT authorship contribution statement

**J. Lebepe:** Conceptualization, Supervision, Writing – review & editing. **N. Khumalo:** Data curation. **A. Mnguni:** Data curation, Writing – review & editing. **S. Pillay:** Data curation, Methodology, Writing – review & editing. **S. Mdluli:** Data curation, Writing – original draft.

## Declaration of Competing Interest

The authors declare that they have no known competing financial interests or personal relationships which have or could be perceived to have influenced the work reported in this article.

## Footnotes

Supplementary material associated with this article can be found in the online version at doi:10.1016/j.dib.2021.107493.
