# Supplementary material for: Data of macroinvertebrates assemblage across different stretches of an urban Palmiet River in Durban, South Africa
Source: Data Brief. 2021 Oct 20;39:107493. doi: 10.1016/j.dib.2021.107493 (PMC8566930; doi:10.1016/j.dib.2021.107493)
Supplement: Supplementary Data S1 [file mmc1.docx]

Appendix 1: A sheet for integrated habitat assessment score (adapted from McMillan 1998)

| **Integrated Habitat Assessment System (IHAS)** | | | | | | |
| --- | --- | --- | --- | --- | --- | --- |
| River Name: | | | | | | |
| Site Name: | | | | Date: |  | |
| SAMPLING HABITAT | 0 | 1 | 2 | 3 | 4 | 5 |
| Stones in current (SIC) | | | | | | |
| Total length (m) of broken water (riffles/rapids) | none | 0-1 | >1-2 | >2-3 | >3-5 | >5 |
| Total length (m) of submerged stones in current (run) | none | 0-2 | >2-5 | >5-10 | >10 |  |
| Number of separate SIC areas kicked | 0 | 1 | 2-3 | 4-5 | 6+ |  |
| Average size (cm) of stones kicked (gravel<2; bedrock>20) | none | <2, >20 | 2-10 | 11-20 | 2-20 |  |
| Amount of stone surface clear (of algae, sediment, silt, etc.) (%) | n/a | 0-25 | 26-50 | 51-75 | >75 |  |
| Protocol: Time (mins) spent actuallly kicking SIC (gravel/bedrock=0) | 0 | <1 | >1-2 | 2 | >2-3 | >3 |
| SIC Scores: (A=SIC boxes total; B=adjustment to equal 20; C=final total) | actual | A | adj. | B | max. 20 | C |
| Vegetation | | | | | | |
| Length (m) of fringing vegetation sampled (banks) | none | 0-½ | >½-1 | >1-2 | 2 | >2 |
| Amount (m2) of aquatic vegetation/algae sampled | none | 0-½ | >½-1 | >1 |  |  |
| Fringing vegetation sampled in: | none |  | run | pool |  | mix |
| Type of veg. (% leafy veg. vs. stems/shoots) (aq. veg. only=49) | none | 0 | 1-25 | 26-50 | 51-75 | >75 |
| Veg Scores: (D=Veg boxes total; E=adjustment to equal 15; F=final total) | actual | D | adj. | E | max. 15 | F |
| Other habitats |  |  |  |  |  |  |
| Stones Out Of Current (SOOC) sampled (m2) (protocol=1m2) | none | 0-½ | >½-1 | 1 | >1 |  |
| Sand sampled (mins) (protocol=1min) (under=present below stones) | none | under | 0-½ | >½-1 | 1 | >1 |
| Mud sampled (mins) (protocol=½min) (under=present below stones) | none | under | 0-½ | ½ | >½ |  |
| Gravel sampled (mins) (protocol=½min) (if all, SIC stone size=<2)* | none | 0-½ | ½ | >½* |  |  |
| Bedrock sampled (all=no SIC/sand/gravel) (if all, SIC stone size=>20)* | none | some |  |  | all* |  |
| Algal presence (1-2m^2^=algal bed; rocks=on rocks; isol.=isolated clumps) | >2m^2^ | rocks | 1-2m^2^ | <1m^2^ | isol. | none |
| Tray identification (using time as per protocol) |  | under |  | correct |  | over |
| Other Habitat Scores: | actual | G | adj. | H | max. 20 | I |
| (G=Other Habitat boxes total; H=adjustment to equal 20; I=final total) |  |  |  |  |  |  |
| HABITAT TOTALS: | | | adj. | J | max. 55 | K |
| (J=total adjustment [B+E+H]; K=Habitat Total [C+F+I]) | | |  |  |  |  |
| Stream condition |  |  |  |  |  |  |
| Physical |  |  |  |  |  |  |
| River make-up (2/3 mix = 2/3 types) | pool |  | run | rapid | 2 mix | 3 mix |
| Average stream width (m) |  | >10 | >5-10 | <1 | 02-Jan | >2-5 |
| Average stream depth (m) | >2 | >1-2 | 1 | >½-1 | ½ | <½ |
| Approximate stream velocity (slow=<½m/s; fast=>1m/s) | still | slow | fast | med. |  | mix |
| Water colour (disc.=visibly discoloured but still clearish) | silty | opaque |  | discol. |  | clear |
| Recent disturbances due to: (constr.=construction) | flood | fire | constr. | other |  | none |
| Bank/riparian vegetation is: (grass=incl. reeds; shrubs=incl. trees) | none |  | grass | shrubs | mix |  |
| Surrounding impacts (erosn.=erosion/shear banks; farm=farmland) | erosn. | farm | trees | other |  | open |
| Anthropogenic litter | absent |  | similar |  | mix |  |
| Anthropogenic litter effect |  |  | severe |  | none |  |
| Left bank cover (%) (rocks and vegetation) | 0-50 | 51-80 | 81-95 | >95 |  |  |
| Right bank cover (%) (rocks and vegetation) | 0-50 | 51-80 | 81-95 | >95 |  |  |
| Stream Condition Total: |  |  |  |  | max. 45 | L |
| Total IHAS Score: (K+L) |  |  |  |  | % |  |
